# Joint Impact of Residential Segregation and Poverty Across the Life Course on Mental Health

**DOI:** 10.1093/geroni/igaf122.1475

**Published:** 2025-12-31

**Authors:** Boeun Kim, Sarah Szanton, Laura Samuel, Karen Bandeen-Roche, Paris Adkins-Jackson, Erik Westlund, Sierra Grey-Coker, Roland Thorpe, Jr.

**Affiliations:** University of Iowa, Iowa City, Iowa, United States; Johns Hopkins University, Baltimore, Maryland, United States; Johns Hopkins University, Baltimore, Maryland, United States; Johns Hopkins Center on Aging and Health, Baltimore, Maryland, United States; Mailman School of Public Health, Columbia University, New York, New York, United States; Johns Hopkins Bloomberg School of Public Health, Baltimore, Maryland, United States; Johns Hopkins University, Baltimore, Maryland, United States; Johns Hopkins University, Baltimore, Maryland, United States

## Abstract

Racially segregated communities often experience concentrated poverty, yet the impact of racialized economic segregation on mental health across the life course remains understudied. This study examined associations between life stage-specific, county-level racialized economic segregation and the number of mentally unhealthy days over the past 30 days among U.S. adults aged 50 and older. Racialized economic segregation during young and middle adulthood was measured using the Index of Concentration at the Extreme (ICE), which quantifies spatial social polarization between deprived (Black individuals in poverty) and privileged (White individuals not in poverty) groups. ICE scores (range: -1 to1) were derived from U.S. Census (1970–2000) and the American Community Survey (2010–2020) data and categorized into quintiles. Life-course addresses were geocoded and linked to county-level data corresponding to their ages. Negative binomial regression models were fitted separately for each life stage and racialized group. This analysis included 365 Black and 885 White participants (56.1% male; 24.3% aged 80+). After adjusting for age, gender, and population density, living in a county in the 4th or 5th ICE quintile during young adulthood —but not middle adulthood—was associated with a 61% or 56% lower likelihood of currently experiencing poor mental health days among White participants (IRR = 0.39, 95% CI: 0.17, 0.91; IRR = 0.44, 95% CI: 0.19, 1.00), compared to those in the 1st quintile. This pattern was not observed among Black participants. This finding contributes to research delineating sensitive periods of exposure to racialized economic segregation and mental health in older adults.

